# Comparison on structural characterization, chain conformation, and neuroprotective activity of polysaccharides extracted from *Albizia julibrissin* Durazz. bark with hot water and ultrasound

**DOI:** 10.1016/j.ultsonch.2025.107608

**Published:** 2025-10-06

**Authors:** Yuanqi Duan, Yanan Liu, Man Li, Yajie Liu, Jiayu Gu, Wei Zhou, Jinfeng Sun, Zhengyu Hu, Mei Jin, Gao Li

**Affiliations:** a Key Laboratory of Natural Medicines of the Changbai Mountain, Ministry of Education, Yanbian University College of Pharmacy, Yanji 133002, PR China; b Department of Pharmacy, Yanbian University Hospital, Yanji 133002, PR China

**Keywords:** *Albizia julibrissin* Durazz., Polysaccharides, Ultrasonic-assisted extraction, Physicochemical properties, Chain conformation

## Abstract

A comparative and multidimensional approach is employed herein to assess how ultrasound affects the extraction yield, structural features, and chain conformation of *Albizia julibrissin* Durazz. bark polysaccharides. The results showed that ultrasound treatment can significantly (P < 0.05) improve the extraction yield, and the optimal extraction process parameters were 50 min, 70 ℃, 30 mL/g, and 201 W. In addition, this study found that ultrasound can alter the chemical composition, characteristic group content, monosaccharide composition molar ratio, molecular weight, and even relative content of glycosidic bond types of *A. julibrissin* bark polysaccharides. Interestingly, the ultrasound treatment followed the midpoint chain scission model and transformed HWE-AJPP1 from a flexible chain conformation to UAE-AJPP1 exhibiting a spherical structure. And the involvement of ultrasound also altered the relatively tight big block-like morphology of HWE-AJPP1, and UAE-AJBP1 showed remarkable thermostability. Compared with HWE-AJBP1, UAE-AJBP1 significantly (P＜0.001) enhanced the viability of injured oxygen glucose deprivation/reoxygenation-induced PC12 cells. And UAE-AJBP1 effectively restored mitochondrial membrane potential, attenuated intracellular ROS levels. The findings of this work establish a solid basis for further exploiting ultrasound-prepared polysaccharides from *A. julibrissin* bark and strongly supports ultrasound’s wider application in polysaccharide-related fields.

## 1 Introduction

*Albizia julibrissin* Durazz., a deciduous shrub in the Fabaceae family, is classified under the genus *Albizia* [1]. Its bark is a traditional Chinese medicinal component targeting neurological disorders, with efficacy in alleviating depression, tranquilizing mind, activating blood and detumescence [2]. *A. julibrissin* bark exhibits a spectrum of pharmacological properties, encompassing antidepressant, anxiolytic, anti-inflammatory, antioxidant, and immune-regulatory effects [2]. While these pharmacological effects are primarily ascribed to triterpenoids, lignans, flavonoids, saponins, and sterols, the exploration of their chemical composition is not comprehensive enough [2]. Kim et al. [3] discovered that aqueous extracts from *A. julibrissin* bark promoted exploratory behavior in the open arms of the elevated plus maze while reducing activity in the closed arms. Jung et al. [4] further elucidated that these extracts substantially increased the binding affinity of [3H]8-hyroxy-2-(di-n-propylamino) tertalin ([3H]8-OH-DPAT) in the frontal cortex and hippocampal CA2/CA3 areas. Despite the fact that polysaccharides are one of the core constituents of the aqueous extracts [5], there has been no systematic investigation into the polysaccharides derived from *A. julibrissin* bark.

The extraction method selected directly impacts the yield, structural features, and functionality of polysaccharides, which are essential for all subsequent experiments [[6], [7], [8]]. Commonly employed extraction approaches for polysaccharides encompass ultrasound-assisted extraction (UAE), microwave-assisted extraction, hot water extraction (HWE), and enzyme-assisted extraction [9]. Among these methods, UAE stands out as a potent approach for polysaccharide acquisition, primarily leveraging the mechanical action, cavitation, and thermal effects of ultrasound to enhance solvent infiltration and polysaccharide solubilization, thus facilitating swift and efficient polysaccharide extraction [[10], [11], [12]]. Compared to traditional extraction methods, UAE has many advantages, such as efficiency, decreased energy and solvent usage, and low operational costs, render it suitable for extensive industrial application [13]. Therefore, UAE is an ideal method for extracting polysaccharides from *A. julibrissin* bark, but it is still unclear how ultrasound may cause changes structural and biological properties in *A. julibrissin* bark polysaccharides.

In recent years, certain researchers have conducted initial investigations into the impact of UAE on the structural characteristics of polysaccharides. Peng et al. [14] discovered that UAE induced alterations in the molar ratio of monosaccharides composition within *Typhonium giganteum* Engl. polysaccharides, and the molecular weight of Peak1 declining from 3191.40 kDa to 2906.425 kDa. Chen et al. [15] reported that UAE increased the content of *α*-1,4-Glc*p*, *α*-1,3,4-Gal*p* and *α*-T-Glc*p* in *Flammulina velutipes* polysaccharides. Wang et al. [16] also found that the microstructure of the polysaccharide UP, extracted via UAE, exhibited larger holes compared to HP. Although these existing studies have confirmed that UAE can affect various structural characteristics of polysaccharides, there are still few reports comparing UAE and HWE to explore how ultrasound affects polysaccharide structure (including their primary and advanced structures). Especially, there is a lack of multidimensional technical comprehensive evaluation, and in-depth analysis of degradation kinetics and chain breakage mechanisms.

On the basis of optimizing the UAE of *A. julibrissin* bark polysaccharides, this study further compared the effects of UAE and HWE on the extraction yield, physicochemical properties, chain conformation, and morphological features of *A. julibrissin* bark polysaccharides using various modern instrument analysis techniques. The degradation kinetics and sugar chain cleavage mechanism of *A. julibrissin* bark polysaccharides were elucidated, and the effectiveness of ultrasound in modifying polysaccharide structure was confirmed. Meanwhile, the potential neuroprotective effect of polysaccharides obtained through UAE was validated through an *in vitro* oxygen glucose deprivation/reoxygenation (OGD/R) model. Overall, this study provides deeper insights into how ultrasound influences polysaccharide structure, facilitating the application of ultrasonic technology in the production of functional foods, pharmaceuticals, and materials.

## 2 Materials and methods

### 2.1 Materials and chemicals

The bark of *Albizia julibrissin* Durazz. was procured from the Bozhou Traditional Chinese Medicine Market situated in Anhui Province, China. Dextran standards with varying molecular weights (certified as national pharmaceutical reference materials) were provided by the China Foods Limited and Drug Control Institute. The subsequent eleven monosaccharide standards, namely rhamnose (Rha), galacturonic acid (GalA), glucose (Glc), arabinose (Ara), fucose (Fuc), galactose (Gal), xylose (Xyl), glucuronic acid (GlcA), ribose (Rib), glucosamine (GlcN), and mannose (Man), were obtained from Shanghai Aladdin Biochemical Technology Co., Ltd, located in Shanghai, China. The PC12 cell line, originating from a highly differentiated rat adrenal pheochromocytoma, was purchased from Shanghai Shangen Biotechnology Co., Ltd. Meanwhile, the reagents required for cell culture were obtained from Sigma-Aldrich, based in St. Louis, MO, USA. Additionally, all other reagents utilized in this investigation were of either HPLC or analytical grade purity.

### 2.2 Single factor experiments and optimized experimental design

An ultrasonic extractor (JM-15D-28, Skymen, China) was employed to refine the UAE procedure for polysaccharides extracted from the bark of *A. julibrissin*. Before the experiment began, the ultrasonic device was calibrated. The impact of varying parameters on the extraction yields of UAE-AJBP was investigated, including extraction time (20, 30, 40, 50, 60 min), temperature (40, 50, 60, 70, 80 ℃), liquid to solid ratio (10, 20, 30, 40, 50 mL/g), and ultrasonic power (100, 150, 200, 250 and 300 W). Apart from alterations in the factors slated for optimization, the other extraction parameters were configured in the following manner: the amount of *A. julibrissin* bark was 5 g, the extraction time was 40 min, the temperature was 70 ℃, the liquid to solid ratio was 20 mL/g, and the ultrasonic power was 150 W. Every experiment was performed with a minimum of three replicates. Based on single-factor experiments, a Box-Behnken response surface design with four factors and three levels was carried out using Design Expert 13.0 software (Stat-Ease Inc., Minneapolis, USA), taking extraction time, temperature, liquid to solid ratio, and ultrasonic power as variables and polysaccharides extraction yield as the evaluation index [17]. A sum of 29 experimental trials were carried out, which included five central points and 24 intermediate points uniformly spread across a spherical area that covered the cube's edges. Subsequently, regression analysis was executed to determine the optimal extraction conditions. Following this, validation trials were conducted under these optimized settings to confirm the precision of the experimental outcomes.

### 2.3 Polysaccharides purification

Polysaccharides were extracted from *A. julibrissin* bark under the optimized conditions of the ultrasonic-assisted extraction method to obtain UAE-AJBP. To compare the effect of ultrasound on the polysaccharide extraction rate and chemical composition, the same conditions as previously mentioned were used, but without applying ultrasound, to extract HWE-AJBP from the *A. julibrissin* bark via hot water extraction. The extraction process was accompanied by concentration of the extract, which was 1/5 of its initial amount. Subsequently, polysaccharide precipitation was induced with 95 % ethanol (final concentration 80 % v/v), and the mixture was incubated overnight at 4 ℃. Then, after undergoing deproteinization through the Sevag method [18] (chloroform/butanol 4:1 v/v), the sample underwent purification on a DEAE-52 cellulose column (2.6 cm × 60 cm) with a 0.3 mol/L NaCl solution at 1 mL/min. We freeze-dried the separated polysaccharide fraction, and then used an 8 kDa retention dialysis membrane for its final purification. Name the samples obtained after dialysis and freeze-drying as UAE-AJBP1 (Purified UAE-AJBP) and HWE-AJBP1 (Purified HWE-AJBP).

### 2.4 Structural characterization

#### 2.4.1 Chemical composition analysis

The total content was determined using the phenol–sulfuric acid method [19], with D-glucose serving as the reference standard and measurements taken via a UV2600 spectrophotometer (Techcomp, China). Protein concentration was evaluated utilizing Bradford's technique [20], employing bovine serum albumin as the standard, whereas the *meta*-hydroxydiphenyl approach [21] was adopted to analyze uronic acid content, calibrated with galacturonic acid.

#### 2.4.2 FTIR analysis

Sample pellets were prepared by mixing with KBr and compressing, followed by FT-IR analysis (Nicolet iS20, Thermo Scientific, USA) at 4 cm^−1^ resolution across 4000–400 cm^−1^ 16 accumulated scans.

#### 2.4.3 Monosaccharide composition analysis

Monosaccharide composition analysis was conducted using high performance liquid chromatography (HPLC) coupled with 1-phenyl-3-methyl-5-pyrazolone (PMP) derivatization [22], employing a Hitachi Primaide system (Japan). Following trifluoroacetic acid (TFA) hydrolysis at 120°C for 3 h, both samples and standards underwent derivatization with PMP (utilizing NaOH and a PMP-methanol solution), were subsequently neutralized with HCl, and then analyzed on a Supersil ODS2 column maintained at 35°C. The mobile phase, consisting of an 83:17 ratio of phosphate buffer to acetonitrile, was pumped at a flow rate of 0.8 mL/min, with detection carried out at 245 nm.

#### 2.4.4 Molecular weight distribution determination

High performance gel permeation chromatography (HPGPC) analysis was conducted on a Shimadzu Bocl104 instrument (Japan) featuring an RID-20A detector to assess molecular weight and homogeneity. The chromatographic separation employed a KS-804 Shodex sugar column (8.0 × 300 mm) with ultrapure water mobile phase flowing at 1 mL/min, at a constant column temperature of 40°C. The molecular weight along with the distribution coefficient were determined through the application of LabSolutions software, which utilized a linear regression equation for the calculations.

#### 2.4.5 NMR spectra analysis

Each polysaccharide fraction was dissolved in D_2_O and subsequently subjected to three rounds of freeze-drying. Following this, the polysaccharide samples were individually dissolved in 0.5 mL of D_2_O along with 20 μL acetone–d6 (internal standard). ^1^H and ^13^C NMR data were subsequently acquired on a 500 MHz Bruker NMR spectrometer (Fällanden, Switzerland).

#### 2.4.6 SEC-MALS analysis

The analysis employing size exclusion chromatography with multi-angle light scattering (SEC-MALS) was carried out utilizing an OHpak guard column in tandem with an SB-805 HQ analytical column (Tosoh Bioscience, Tokyo, Japan), following the method described by [23], with appropriate modifications. The mobile phase comprised 0.1 mol/L NaNO_3_ supplemented with 0.01 % NaN_3_, and the flow rate was kept constant at 0.4 mL/min throughout the procedure. The sample, with a concentration of 1 mg/mL, was dissolved using the mobile phase and allowed to stand undisturbed overnight. Subsequently, it underwent centrifugation at 10000 g for a duration of 40 min. After that, the supernatant was passed through a 0.45 μm membrane filter, and 500 μL of the filtrate was introduced into the analytical system. The system underwent calibration employing a dextrose standard, during which the refractive index increment (dn/dc) was adjusted to a value of 0.138. Data collection and subsequent analysis were carried out utilizing ASTRA software (Wyatt Technology, USA).

#### 2.4.7 Microscopic morphology observation

The examination of the polysaccharides' surface morphology was performed utilizing a scanning electron microscope (SEM, Gemini SEM 360, ZEISS, Oberkochen, Germany). Dried powder samples were affixed to stubs using conductive adhesive, coated with gold via sputtering, and then placed in the SEM for analysis.

The morphology features of the polysaccharides were investigated employing a Bruker NanoScope Dimension Icon atomic force microscope (Germany). Following dilution to 2.5 µg/mL, 5 µL aliquots were transferred onto freshly mica sheets, air-dried, and then imaged in tapping mode.

#### 2.4.8 TGA analysis

Thermal analysis was performed on 15 mg polysaccharide samples using a NETZSCH TG 209 F1 Libra thermogravimetric analyzer (Selb, Germany) with the following parameters: temperature range 30-800°C, heating rate 20°C/min, and nitrogen flow rate 50 mL/min.

#### 2.4.9 Triple helix structure and CD spectroscopy analysis

In line with a recent study [24], the triple helix structure of each fraction was determined through the Congo red assay, with a UV–vis spectrophotometer employed to record the maximum absorption wavelength (λmax) spanning from 200 to 800 nm. Additionally, the circular dichroism (CD) spectra of the polysaccharide solutions were recorded utilizing a J-180CD spectrometer (JASCO, Japan) over a wavelength spectrum of 190 to 320 nm, at a scanning speed of 200 nm/min. The resulting data were then averaged and smoothed prior to analysis.

### 2.5 Biological activity

The cultivation of PC12 cells was carried out using Dulbecco's Modified Eagle's Medium (DMEM) supplemented with 10 % fetal bovine serum, 100 U/mL penicillin, and 100 μg/mL streptomycin. Subsequently, these cells were placed in an incubator at 37°C with a 5 % CO_2_ atmosphere, and the culture medium was replaced every 48 h. At 80–90 % confluency, cells were trypsinized (0.25 %), centrifuged (1000 rpm, 5 min), and reseeded in fresh medium. For OGD/R modeling, some modifications have been made according to reference [25]. Firstly, pre-treated the cells with polysaccharide samples for 2 h, followed by OGD/R induction. PC12 cells were transferred to glucose-free DMEM and placed in a hypoxic chamber for 9 h (1 % O_2_, 5 % CO_2_ and 94 % N_2_) to simulate ischemia. After hypoxia, the cells were returned to normoxia (5 % CO_2_, 95 % air) and reoxygenated in high-glucose DMEM for 24 h. The PC12 cells in the control groups were cultured with normal medium conditions. 24 h after the model was constructed, the assessment of cell viability was conducted using the CCK-8 assay. Specifically, 10 μL of the reagent was introduced into each well, followed by an incubation period lasting 1 to 2 h. And, the absorbance was determined at a wavelength of 450 nm by employing a microplate reader (Infinite 200 PRO, Tecan, Switzerland).

The Mitochondrial membrane potential (△ψm) was assessed using JC-1 kit (Beyotime, C2006, China) following manufacturer's instructions. Briefly, treated PC12 cells in 96-well plates were incubated with JC-1 (10 μg/mL in serum-free medium, 1:500 dilution) at 37°C for 30–60 min, followed by PBS washing. Fluorescence was quantified microscopically, with △ψm calculated as the red/green ratio using Image J software [26]. Intracellular ROS levels were evaluated using DCFH-DA (Beyotime, S0033S, China). Treated PC12 cells were incubated with the probe in serum-free medium at 37°C for 30 min, washed twice with PBS, and analyzed using a microplate reader (Infinite M200 PRO; excitation 488 nm, emission 525 nm). Fluorescence intensity was quantified with Image J software [26].

### 2.6 Statistical analysis

The required results were obtained by repeating the experiment three times per group, and the results were expressed as mean ± SD. Statistical analyses were conducted using one-way ANOVA with multiple comparison tests in GraphPad Prism 8.0 software. For ANOVA results, statistical significance was defined as P < 0.05, with significance levels denoted as *P < 0.05, ^**^P < 0.01, and ^***^P < 0.001.

## 3 Results and discussion

### 3.1 Optimization of ultrasound-assisted extraction conditions

#### 3.1.1 Single-factor experiment

**Fig. 1:**
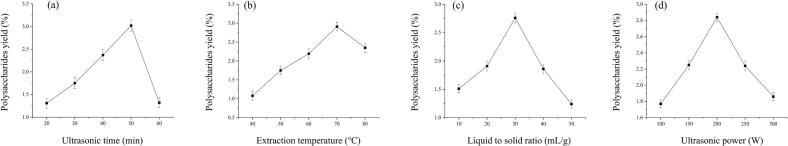
Effect of different independent factors on the extraction yield of *A. julibrissin* bark polysaccharides.

Key process variables including ultrasonic time (X_1_; min), extraction temperature (X_2_; °C), liquid to solid ratio (X_3_; mL/g), and ultrasonic power (X_4_; W) were independently optimized for *A. julibrissin* bark polysaccharide extraction using single-factor methodology. The correlation between AJBP yield (y-axis) and individual factors adjusted across different gradients (x-axis) was illustrated in Fig. 1.

Maximum UAE-AJBP production was achieved at 50 min of ultrasonic treatment (Fig. 1a), with the yield showing an increasing trend up to this point and decreasing thereafter in the tested time (20–60 min). The initial increase was attributed to strengthened cavitation and thermal effects that enhanced polysaccharide solubility. However, when ultrasonication exceeded 50 min, the yield decreased, which could be explained by the intense mechanical shearing action of ultrasound, prolonged sonication might lead to damage to the polysaccharide structure [27]. Fig. 1b demonstrated that UAE-AJBP yield was lowest at 40°C but increased significantly with rising temperature, peaking at 70°C. This improvement probably resulted from enhanced polysaccharide solubility at elevated temperatures [28]. Once the temperature surpassed 70°C, a decrease in yield was observed, likely attributable to structural modifications induced by elevated temperatures, as referenced in [28]. Fig. 1c illustrated that the yield of UAE-AJBP exhibited an upward trend as the liquid to solid ratio was adjusted from 10:1 to 30:1 mL/g, possibly due to the augmented concentration gradient of polysaccharides established between the plant tissue and the solvent. This intensified gradient promotes more rapid diffusion and dissolution, thereby resulting in a higher extraction yield. Nevertheless, as the ratio was escalated to 50:1 mL/g, a decline in yield was noted. This phenomenon might stem from the fact that an increased solvent volume reduces the ultrasonic energy per unit volume, consequently leading to a decreased carbohydrate content [29].

The extraction efficiency exhibited a positive correlation with ultrasonic power levels between 100–300 W (Fig. 1d). This phenomenon primarily arises from the mechanical action of ultra-high pressure. The ultra-high pressure enables the solvent to more effectively penetrate into cellular substances, disrupts the integrity of cells, and modifies the tissue's structure. Such alterations improve the transfer of the desired extract into the solvent within the cell. Notably, the polysaccharide yield reached a maximum at 200 W but subsequently decreased with further power escalation. This decrease may be attributed to intensified mechanical stress from elevated power levels, which triggers progressive cellular disruption via cavitation effects, ultimately creating potential barriers that hinder polysaccharide liberation [30]. From the single-factor experiments, the following optimal conditions emerged and were adopted: 50 min, 70°C, 30 mL/g, and 200 W.

#### 3.1.2 Response surface for ultrasound-assisted extraction

Drawing on the outcomes of the single-factor experiments, a Box-Behnken design (BBD) incorporating four factors at three distinct levels was utilized [31]. The collected experimental data underwent analysis and were fitted to derive the subsequent regression equation:

(1) $$Y=3.40-0.0475X_{1}-0.0700X_{2}-0.0700X_{3}+0.0275X_{4}+0.0550X_{1}X_{2}+0.0025X_{1}X_{3}+0.0100X_{1}X_{4}+0.0700X_{2}X_{3}-0.2600X_{2}X_{4}-0.3425X_{3}X_{4}-0.9424X_{1}^{2}-0.8912X_{2}^{2}-1.08X_{3}^{2}-1.37X_{4}^{2}$$

**Fig. 2:**
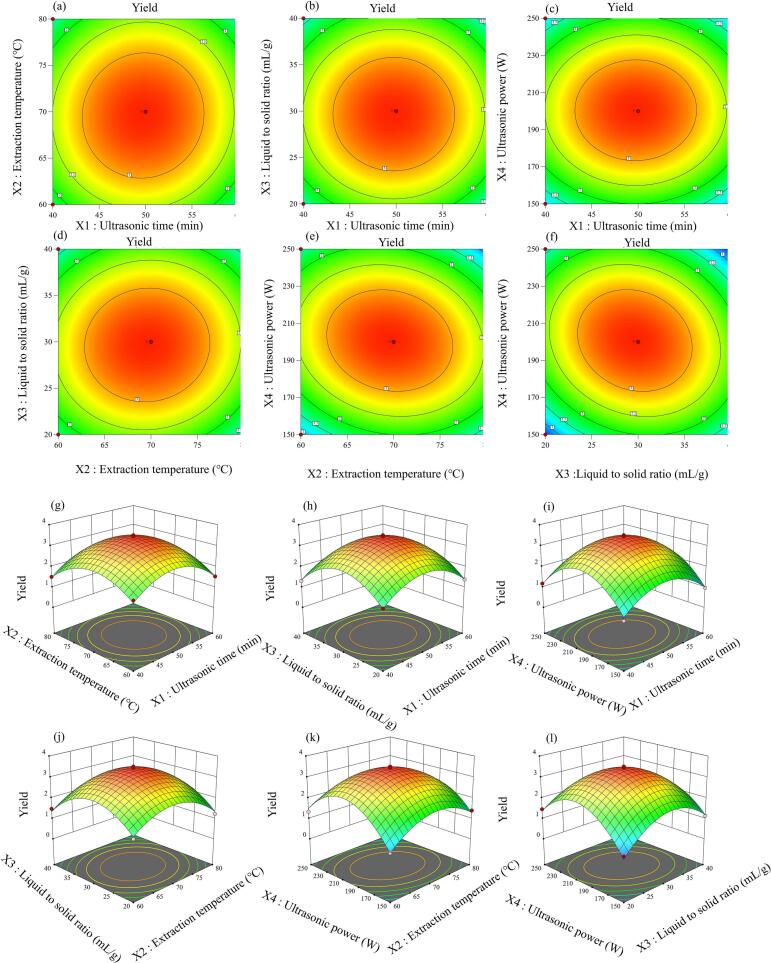
Response surfaces plots (a-f) and contour plots (g-l) of the interactive effects on the *A. julibrissin* bark polysaccharides yield.

**Fig. 3:**
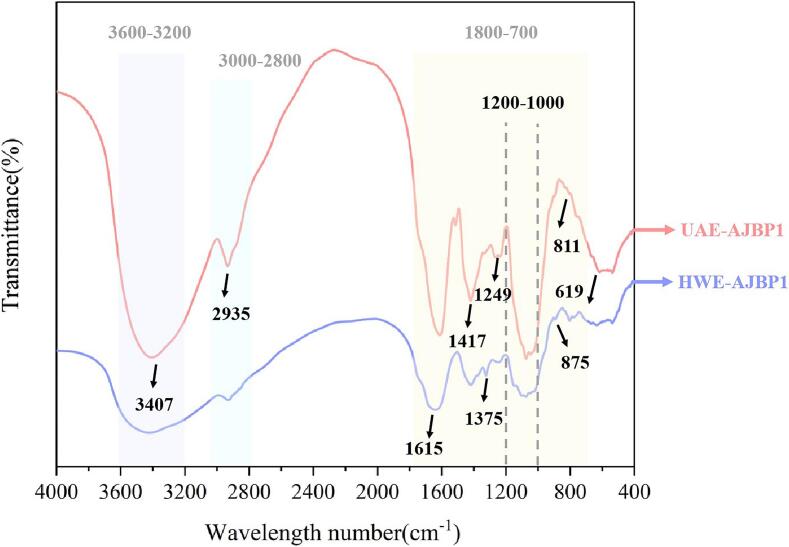
FT-IR spectra of HWE-AJBP1 and UAE-AJBP1.

As presented in Table 1, the regression model exhibited a notably high level of significance (P < 0.0001) concerning the yield of UAE-AJBP, suggesting that the model was appropriately constructed. Moreover, the model attained a high R^2^ value of 0.9891, which signifies an excellent fit and effective portrayal of the data. And, the R^2^_adj_ value stood at 0.9782, indicating a robust relationship between the actual and forecasted values. The experimental findings validated the model's accuracy in predicting UAE-AJBP production with considerable precision, thus serving as a valuable tool for process improvement. 2D contour maps (Fig. 2a–f) and 3D response surface plots (Fig. 2g–i) provided clear insights into the optimal values of individual factors and their interrelationships. A steeper slope in the 3D plot indicated a more pronounced effect of the factor, whereas an increasingly elliptical contour in the 2D map signifies the stronger interaction between factors [17]. From this, it can be seen that the results of the analysis of variance in Fig. 2 were consistent with those in Table 1, which means that the interaction between factors X_2_X_4_ and X_3_X_4_ was more significant. And, every image depicted in Fig. 2g–i exhibited openings facing downward, which substantiates that the model achieved the most favorable extraction circumstances and delivered the highest yield. These findings underscored the dependability of the devised model. Additionally, the model predicted the optimal extraction conditions as follows: 49.735 min, 69.565°C, 29.635 mL/g, and 200.939 W, and the predicted maximum yield was 3.408 %. In order to enhance the feasibility of applying the experimental approach on an industrial scale, the optimal parameters were slightly adjusted to 50 min, 70°C, 30 mL/g, and 201 W. Following this modification, three replicate experiments were carried out under these revised conditions, yielding an optimal extraction rate for UAE-AJBP of 3.16 ± 0.19 %.

**Table 1:** ANOVA for response surface quadratic model of the UAE-AJBP yield.

**Source**	**Sum of squares**	**Df**	**Mean square**	***F*-value**	***P*-value**
Model	21.08	14	1.51	90.64	< 0.0001^⁎⁎⁎^
X_1_	0.0271	1	0.0271	1.63	0.2225
X_2_	0.0588	1	0.0588	3.54	0.0809
X_3_	0.0588	1	0.0588	3.54	0.0809
X_4_	0.0091	1	0.0091	0.5463	0.4721
X_1_X_2_	0.0121	1	0.0121	0.7284	0.4078
X_1_X_3_	0.0000	1	0.0000	0.0015	0.9696
X_1_X_4_	0.0004	1	0.0004	0.0241	0.8789
X_2_X_3_	0.01960	1	0.01960	1.18	0.2957
X_2_X_4_	0.2704	1	0.2704	16.28	0.0012^⁎⁎^
X_3_X_4_	0.4692	1	0.4692	28.24	0.0001^⁎⁎^
X_1_^2^	5.76	1	5.76	346.78	< 0.0001^⁎⁎⁎^
X_2_^2^	5.15	1	5.15	310.09	< 0.0001^⁎⁎⁎^
X_3_^2^	7.62	1	7.62	458.52	< 0.0001^⁎⁎⁎^
X_4_^2^	12.22	1	12.22	735.43	< 0.0001^⁎⁎⁎^
Residual	0.2326	14	0.0166		
Lack of fit	0.2085	10	0.0280	3.46	0.1216
Pure error	0.0241	4	0.0060		
Correlation total	21.31	28			
*R*^2^	0.9891		*R*^2^_Adj_	0.9782	
*C*.*V*.%	7.91		Pred R-Squared	0.9419	

⁎*P* < 0.05. ⁎⁎*P* < 0.01. ⁎⁎⁎*P* < 0.001.

To evaluate the effect of ultrasound treatment on the extraction rate of polysaccharides from *A. julibisen* bark, the extraction rates were compared using ultrasound-assisted extraction and hot water extraction, respectively. That is to say, the extraction parameters for hot water extraction of *A. julibrissin* bark polysaccharides (HWE-AJBP) were as follows: 70 °C, 50 min and 30:1 mL/g liquid solid ratio. Make all experimental parameters of HWE-AJBP the same as those of UAE-AJBP, except for the application of ultrasound. Notably, the extraction yield of HWE-AJBP was 0.64 ± 0.13 %, which was about 2 % lower than that of UAE-AJBP. This primarily stemmed from the inherent physical characteristics of ultrasound, which facilitate the liberation and solubilization of polysaccharides [32]. Specifically, the mechanical action of ultrasound disrupts the cell wall structure, thereby augmenting intracellular mass transfer processes [33]. On the other hand, during cavitation, the cavitation state may shift from stable to transient cavitation as a result of the synergistic influence of both cavitation and thermal effects. In this transient state, energy absorption leads to the creation of an environment characterized by high temperature and pressure, which prompts the instantaneous rupture of cell walls and consequently enhances the extraction efficiency [34]. These findings confirm the crucial role of ultrasound in achieving maximum extraction yield and highlight its high efficiency in the field of polysaccharide extraction.

### 3.2 Structural characterization

#### 3.2.1 Chemical composition analysis of UAE-AJBP and HWE-AJBP

It is well-recognized that the extraction techniques employed for extracting polysaccharides from plant sources can affect their physicochemical characteristics [35]. For this purpose, we examined how different extraction methods, namely UAE and HWE, influence the properties of the obtained polysaccharide components. As presented in Table 2, notable disparities were observed in the chemical composition between UAE-AJBP and HWE-AJBP, which were derived using the two distinct extraction methods.

**Table 2:** The chemical composition of UAE-AJBP, HWE-AJBP, UAE-AJBP1 and HWE-AJBP1, and the monosaccharide composition of UAE-AJBP1 and HWE-AJBP1.

	Total sugar (%)	Protein (%)	Uronic acid (%)	Monosaccharide composition (molar ratio)							
				Man	Rha	GlcA	GalA	Glc	Gal	Xyl	Ara
UAE-AJBP	27.51 ± 0.57	7.48 ± 0.36	6.18 ± 0.41	/	/	/	/	/	/	/	/
HWE-AJBP	20.55 ± 0.64	10.51 ± 0.57	2.69 ± 0.62	/	/	/	/	/	/	/	/
UAE-AJBP1	90.76 ± 0.49	–	9.37 ± 0.54	10.46	20.01	2.61	8.37	34.22	40.83	1.00	16.24
HWE-AJBP1	91.97 ± 0.69	–	4.85 ± 0.40	5.25	10.43	2.39	4.16	70.21	16.14	1.00	16.35

“–” was not detected.

The data comparison in Table 2 indicated that the total sugar content of UAE-AJBP was markedly higher (P < 0.05), measuring 27.51 ± 0.57 %, compared to 20.55 ± 0.64 % for HWE-AJBP. This suggested that ultrasonic treatment enhanced the total sugar content while simultaneously decreasing impurity levels. The extraction efficiency of total sugars from plant-derived polysaccharides is greatly affected by how effectively the cell wall and polysaccharides are dissolved, as cavitation can expedite cell wall destruction, thereby facilitating polysaccharide dissolution and release [36]. Similarly, this may also be associated with the cleavage of polysaccharide glycosidic linkages during ultrasonic processing [37].

During the process of polysaccharide extraction, it is common to encounter impurities including inorganic salts, proteins, pigments, as well as small-molecular substances [38]. Generally speaking, the protein content is relatively high, mainly due to the nature of the extraction method and the complex composition of *A. julibrissin* bark [38]. In this study, the protein content in UAE-AJBP is notably lower (P < 0.05) compared to that in HWE-AJBP. This observation could be attributed to the disruption of the chemical bonding force linking polysaccharides and proteins under ultrasonic influence. This disruption promotes the separation between polysaccharides and proteins, which in turn improves the solubilization efficiency of polysaccharides [39]. Ultimately, the objective of this process is to elevate polysaccharide content while concurrently decreasing protein content. One notable point is that when protein content decreases, it doesn't merely improve the solubility of polysaccharides, it also makes it easier for polysaccharide molecules to pass through biological membranes, potentially amplifying their pharmacological actions as a result [40]. This characteristic makes UAE-AJBP1 more promising in fields such as food, drug delivery, and drug carriers [40]. In other words, relying exclusively on high temperatures to disrupt cell walls and attain efficient extraction of polysaccharides with high purity is far from adequate. Ultrasonic-assisted extraction method is a better choice than hot water extraction method for extracting *A. julibrissin* bark polysaccharides.

Interestingly, the uronic acid content of UAE-AJBP obtained by ultrasound treatment was also higher than that of HWE-AJBP, at 6.18 ± 0.41 % and 2.69 ± 0.62 %, respectively. This may be attributed to the rupture of polysaccharide chains due to ultrasonic treatment, as well as a rise in free uronic acid originating from polysaccharides [41]. Polysaccharides with elevated uronic acid proportions possess a negative charge owing to their distinct electrical attributes, potentially influencing their activity. These polysaccharides dissolve in water through hydrogen bonding, adopting a relatively dispersed state [36,42]. This dispersed condition may potentially be more favorable for demonstrating the functional characteristics of polysaccharides, and it could possibly augment their stability within biological fluids. These results were consistent with the observations made in the research carried out by Sun et al. [41], Li et al. [43], and Mansour et al. [44], which showed that ultrasonic processing can increase the content of total sugar and uronic acid, while simultaneously reducing the protein content. In summary, the variances in the chemical characteristics of *A. julibrissin* bark polysaccharides obtained indicated that UAE was shown to be a more effective method compared to HWE.

To delve deeper into examining how ultrasound treatment impacts the primary structure, chain conformation, and functional characteristics of polysaccharides, and to minimize the interference of other factors on the analytical outcomes as much as feasible, Sevag method and DEAE-52 cellulose column (NaCl, 0.3 mol/L) were used in the experimental method to purify and separate UAE-AJBP and HWE-AJBP, and high-purity polysaccharide fractions UAE-AJBP1 and HWE-AJBP1 were obtained successively (with total sugar content higher than 90 %), and protein interference was completely eliminated by Sevag method.

#### 3.2.2 FTIR analysis of UAE-AJBP1 and HWE-AJBP1

Analysis of the effect of ultrasonic treatment on polysaccharide characteristic groups by fourier transform infrared spectrometer (FTIR). FTIR, characterized by its rapidity, non-destructiveness, and accessibility as a spectral technique, has found extensive application in the analysis of polysaccharide structures [45]. It functions as an effective approach for analyzing the types of glycosidic bonds, identifying functional groups, and determining the configuration of glycan rings present in polysaccharides [27].

The findings were categorized into three distinct characteristic regions: the O-H stretching band (ranging from 3600 to 3200 cm^−1^), the C-H stretching band (spanning 3000 to 2800 cm^−1^), and the fingerprint region (covering 1800 to 700 cm^−1^) [46]. The FTIR spectra of *A. julibrissin* bark polysaccharides, both with and without ultrasonic treatment, displayed the classic characteristic peaks of polysaccharides within the 4000–400 cm^−1^ range, as shown in Fig. 3. Specifically, the characteristic absorption peaks around 3407 and 2935 cm^−1^ were attributed to the vibrations of O-H and C-H bonds, respectively. The absorption bands observed around 1615 and 1417 cm^−1^ were attributed to the absorption of the deprotonated carboxylic group, thereby verifying the presence of uronic acid [47,48], and these observations were consistent with the measured uronic acid content. A peak was observed at 1375 cm^−1^, potentially attributable to the symmetric stretching vibration of the C=O bond [29]. A weak peak at 1249 cm^−1^ was noted, likely corresponding to variable angle vibrations of the C-H bond [48]. Additionally, the presence of multiple peaks within the 1200–1000 cm^−1^ range for both UAE-AJBP1 and HWE-AJBP1 indicated the existence of glycosidic linkages and pyranose rings in the polysaccharide structure. And the absorption bands detected at 875 and 811 cm^−1^ suggested that both UAE-AJBP1 and HWE-AJBP1 comprised α-glycosidic as well as β-glycosidic linkages. The absorption peak at 619 cm^−1^ was linked to the deformation vibration of C-C or C-H bonds within cyclic configurations [49]. These cyclic structures contribute to shaping the three-dimensional architecture of the polysaccharides, which in turn impacts their interactions with biological receptors in the body and subsequently influences their biological activity [50].

It was readily apparent from Fig. 3 that the infrared spectra of UAE-AJBP1 and HWE-AJBP1 exhibited a high degree of similarity, with no discernible differences in the absorption bands between polysaccharides that were untreated and those subjected to ultrasound treatment. These findings demonstrated that ultrasonic treatment left the principal functional groups of polysaccharides unchanged. As reported by Yu et al. [36], Liu et al. [51], and Zhang et al. [52], no notable discrepancies were found in the characteristic groups extracted polysaccharides via HWE and UAE. Our findings align with the previously mentioned studies. Nonetheless, according to the Beer-Lambert principle, there exists a direct relationship between the absorption intensity of an analyte and its concentration [45]. In theory, the quantification of polysaccharide components could be achieved by analyzing the intensity of their unique absorption bands. Therefore, the varying peak adsorption intensities of them suggested disparities in their structures, potentially giving rise to differences in their biological functions.

#### 3.2.3 Monosaccharide composition analysis of UAE-AJBP1 and HWE-AJBP1

Monosaccharides, essential units of polysaccharides, link via glycosidic bonds to form diverse chains and branches, which crimp and fold into polysaccharides with unique architectures and bioactivities [53]. It can modify the chain length, functional group, spatial configuration, charge characteristics, and other structural parameters, consequently affecting their rheological behavior, viscoelastic properties, solubility, stability, and biological activity [54,55]. Moreover, monosaccharide molar ratio variations in polysaccharides imply differences in glycosidic bonds and linkages, influencing functionality. Thus, studying monosaccharide composition and molar ratios is vital for revealing polysaccharide structures and biofunctions, probing ultrasonic treatment mechanisms, and ensuring functional polysaccharide quality control [56], providing a theoretical basis for future research on polysaccharides from *A. julibrissin* bark.

**Fig. 4:**
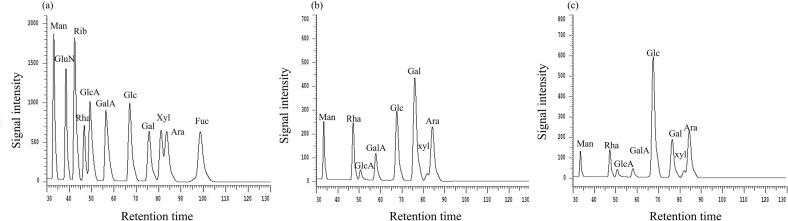
Monosaccharide composition of UAE-AJBP1 (a) and HWE-AJBP1(b).

The monosaccharide composition analysis results of UAE-AJBP1 and HWE-AJBP1 were shown in Fig. 4 and Table 2. Interestingly, this research indicated that both UAE-AJBP1 and HWE-AJBP1 comprised the same eight monosaccharides. Additionally, Gal, Glc, and Rha were the primary monosaccharides in UAE-AJBP1, while Glc, Ara, and Gal were the most abundant in HWE-AJBP1. And, the molar ratios of Man, Rha, GlcA, GalA, Glc, Gal, Xyl and Ara of UAE-AJBP1 were 10.46: 20.01: 2.61: 8.37: 34.22: 40.83: 1.00:16.24, while the molar ratios of HWE-AJBP1 were 5.25: 10.43: 2.39:4.16: 70.21: 16.14: 1.00: 16.35. The findings demonstrated that while the monosaccharide composition remained unchanged across different extraction techniques, these methods had the potential to impact the molar ratio of monosaccharides. One possible explanation is that the mechanical bond breaking and cavitation effects of ultrasound accurately identify the degradation phenomenon caused by the glycosidic bond positions of polysaccharides. This occurrence is probably attributable to the ultrasonic-induced breakdown of polysaccharide chains and the disruption of intermolecular hydrogen bonding. These impacts result in a decrease in both molecular weight and the flexibility of the polymer chains, as they weaken hydrogen bonding and van der Waals forces, consequently altering the monosaccharide composition molar ratios of the polysaccharides [57]. The findings from this investigation were highly consistent with those documented in earlier studies [58], suggesting that although ultrasonic treatment did not change the particular types of monosaccharides involved, it did cause a variation in the relative content of each monosaccharide component. Meanwhile, due to the intervention of ultrasound, the content of Man, Rha, GalA, and Gal has almost doubled. According to the structure–activity relationship reports in relevant reviews, the combination of high levels of Man, Rha, GalA, and Gal endows polysaccharides with better pharmacological effects, implying that UAE-AJBP1 has more development and utilization advantages in the field of biomedicine [[59], [60], [61]].

#### 3.2.4 Molecular weight distribution determination and degradation kinetics analysis

Molecular weight (Mw) correlates clearly with polysaccharide physicochemical properties, greatly affecting their characteristics and functions [62]. Evidently, understanding the link between Mw and polysaccharide properties holds substantial importance, and exploring the influence of ultrasound on polysaccharide structural characteristics on this basis is also of profound significance. Polysaccharides with different Mw show distinct functions, influencing their applications. Generally, plant polysaccharides with a high Mw typically possess larger molecular dimensions, which hinders their ability to traverse the cell membrane and express their biological activities. While low Mw polysaccharides may bind to active sites, they often fail to form complex spatial structure [55]. Studying plant polysaccharides of varying Mw helps identify the range with superior activity, which is vital for future research on *A. julibrissin* bark polysaccharides' structure–activity relationship and ultrasound's effects on their properties and chain conformations.

**Fig. 5:**
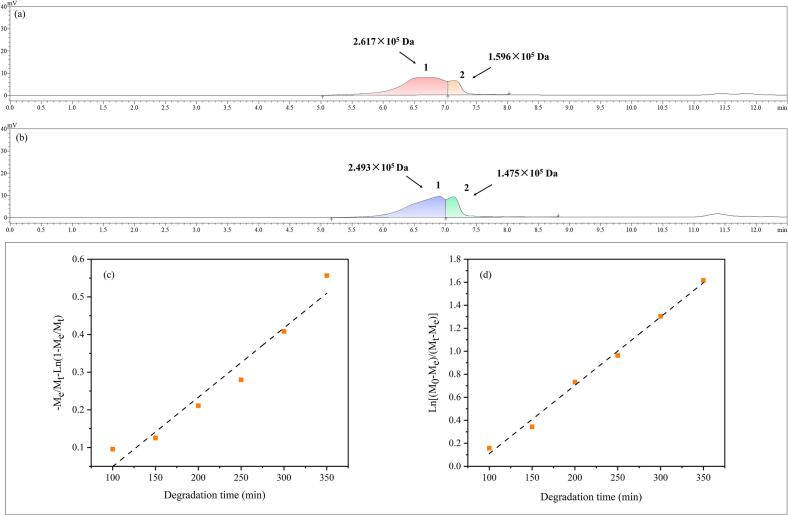
Molecular weight distribution by HPGPC: HWE-AJBP1 (a) and UAE-AJBP1 (b); Degradation kinetics of UAE-AJBP1: the random chain scission model (c) and the midpoint chain scission model (d).

The Mw distributions of UAE-AJBP1 and HWE-AJBP1 were depicted in Fig. 5a–b and summarized in Table 3. It can be seen that both UAE-AJBP1 and HWE-AJBP1 exhibit two peak shapes, signifying the presence of two kinds of polysaccharide types with varying Mw. Each peak represents a distinct Mw range, indicating that both UAE-AJBP1 and HWE-AJBP1 were heterogeneous polysaccharides. Notably, Peak 1 exhibited the highest Mw, measuring 2.493 × 10^5^ Da for UAE-AJBP1 and 2.617 × 10^5^ Da for HWE-AJBP1. Meanwhile, the Mw values for Peak 2 were 1.475 × 10^5^ Da and 1.569 × 10^5^ Da for UAE-AJBP1 and HWE-AJBP1, respectively. The findings indicated that for both Peak 1 and Peak 2, HWE-AJBP1 exhibited a higher Mw compared to UAE-AJBP1, suggesting that the Mw of the polysaccharide fraction (HWE-AJBP1) derived from HWE was larger than that obtained through ultrasonic treatment. According to literature reports, a smaller Mw range could promote the development and application of polysaccharides in the fields of food, medicine, and cosmetics, which also implies that UAE-AJBP1 has greater potential for development [63]. In addition, the difference in molecular weight between UAE-AJBP1 and HWE-AJBPI suggested that ultrasound treatment had an impact on the structural properties of these polysaccharides. These results were in accordance with *Typhonium giganteum* Engl. Polysaccharides [14], *Laminaria japonica* polysaccharides [64] and *Artemisia argyi* leaves polysaccharides [36]. This difference may be ascribed to ultrasonic treatment, which modifies the glycosidic bonds and Mw distribution of polysaccharides. Such shear forces generally facilitate the dispersion of polysaccharide aggregates, thereby inducing the degradation of polysaccharide molecules [44]. Ultrasonic degradation mainly depends on intense and rapidly varying mechanical motion to break down polysaccharides in a medium featuring high-speed wave vibrations and shear forces. This mechanism results in a decrease in molecular weight by cleaving susceptible chemical bonds [55]. Furthermore, the polydispersity index (Mw/Mn) functions as a metric for assessing the Mw distribution of polymers, with values closer to 1 indicating a more uniform Mw distribution. As was shown in Table 3, the polydispersity indices of UAE-AJBP1 peak 1 and peak 2 were 1.12 and 1.06, respectively, while the polydispersity indices of HWE-AJBP1 peak 1 and peak 2 were 1.10 and 1.04, respectively. The heightened polydispersity is presumably due to the significant cleavage of large molecular chains throughout the degradation process, coupled with a marked increase in fragments with low Mw. Moreover, the complex structural characteristics of polysaccharides and the selective action of ultrasonic degradation contributed to non-uniform degradation pathways [57]. Ulteriorly, to explore the selectivity of ultrasonic degradation regarding polysaccharide structure and the mechanism of chain scission, an investigation into ultrasonic degradation kinetics was conducted.

**Table 3:** Molecular weight distribution of UAE-AJBP1 and HWE-AJBP1.

Name	Peaks	Peak area percentage (%)	Molecular weight (Da)	M_W_/M_n_
UAE-AJBP1	1	81.21 %	2.493 × 10^5^	1.12
	2	18.78 %	1.475 × 10^5^	1.06
HWE-AJBP1	1	82.26 %	2.617 × 10^5^	1.10
	2	17.73 %	1.596 × 10^5^	1.04

Given the polydisperse nature of most polymers, obtaining insights into the chain scission region and the Mw dependent rate coefficient is crucial for accurately assessing degradation kinetics (with Peak 2 serving as a representative example) [65]. In order to acquire a more profound understanding of the degradation mechanism of UAE-AJBP1 and the particular impact of ultrasound on the cleavage of glycosidic bonds, the well-established Schmid model, as utilized in previous research, was applied to mimic the random and midpoint chain breakage processes triggered by ultrasonic treatment [66]. As depicted in Fig. 5c–d, the random scission model (Fig. 5c) exhibited a scattered situation, whereas the midpoint scission model's (Fig. 5d) diagram demonstrated more better linear relationship (R^2^ > 0.98), suggesting that the midpoint chain scission model provided a more suitable description of UAE-AJBP1′s ultrasonic degradation behavior. A plausible explanation lies in the tendency for collapsing cavitation bubbles to form in proximity to the *A. julibrissin* bark polysaccharides's center of gravity. Thus, it can be reasonably inferred that the polysaccharide chains of UAE-AJBP1 may undergo ordered cleavage in the middle segment of the chain backbone, as opposed to random fragmentation.

#### 3.2.5 NMR spectra analysis

In the field of polysaccharide structure research, nuclear magnetic resonance spectroscopy (NMR) technology has become an indispensable analytical tool for the primary structure of polysaccharides, especially demonstrating unique advantages in analyzing the molecular structure of complex polysaccharides [67]. NMR data can be used to analyze the glycosidic bond configuration, glycosidic bond connection mode, sugar types, as well as backbone substitution pattern of polysaccharides through computational processing, which can eliminate various complex chemical degradation processes compared to other methods. It should be noted that when conducting solution-state NMR studies on polysaccharides, the sample preparation process holds significant importance. In particular, the use of a “pure” polysaccharide sample is strongly advised for accurate determination. Following this, the protons within the polysaccharide structure are typically exchanged with deuterium atoms. This exchange is commonly achieved through a series of solubilization/lyophilization cycles performed in deuterium oxide (D_2_O), with the cycle being repeated three times. The rationale behind this approach is that the hydrogens bonded to oxygens in hydroxyl groups undergo rapid exchange with the solvent, resulting in their absence of detectable signals in NMR spectroscopy. Consequently, this reduces signal density and the likelihood of resonance overlap, thereby facilitating easier peak identification and structural elucidation [68]. In this study, NMR technology was mainly used to determine whether ultrasound treatment affected the glycosidic bond configuration and type of polysaccharides from *A*. *julibrissin* bark.

**Fig. 6:**
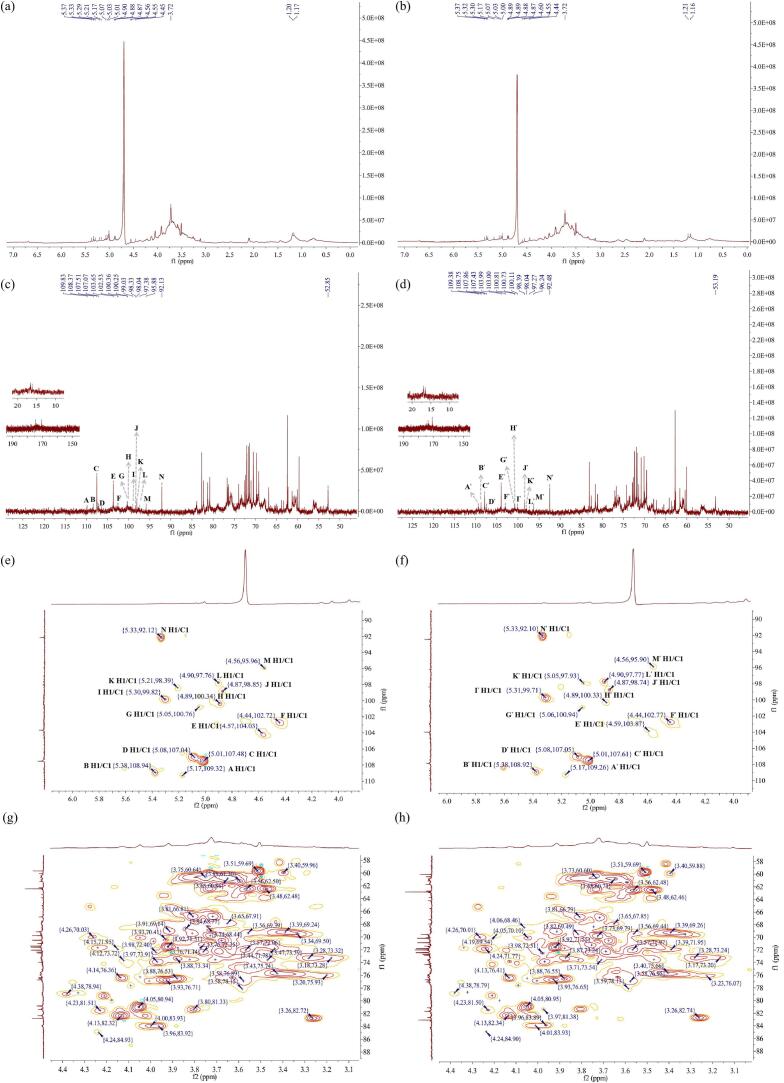
^1^H NMR spectrum of HWE-AJBP1 (a), ^1^H NMR spectrum of UAE-AJBP1 (b); ^13^C NMR spectrum of HWE-AJBP1 (c), and ^13^C NMR spectrum of UAE-AJBP1 (d); HSQC NMR spectrum of HWE-AJBP1 *δ*_H1/C1_ cross-peaks (e), HSQC NMR spectrum of UAE-AJBP1 *δ*_H1/C1_ cross-peaks (f); Enlarged drawing of *δ*_H2-H6/C2-C6_ cross-peaks in HSQC-NMR spectrum of HWE-AJBP1 (g), Enlarged drawing of *δ*_H2-H6/C2-C6_ cross-peaks in HSQC-NMR spectrum of UAE-AJBP1 (h).

The spectra of UAE-AJBP1 and HWE-AJBP1 were documented, including ^1^H NMR, ^13^C NMR and HSQC analysis. In the process of determining the chemical shifts of UAE-AJBP1 and HWE-AJBP1, a calibration procedure was carried out. The ^1^H signals were calibrated against the resonance of D_2_O (*δ*_H_ = 4.70 ppm), while the ^13^C signals were calibrated using acetone-D_6_ as the reference (*δ*_C_ = 29.84 ppm) [69]. In the ^1^H NMR spectrum (Fig. 6a–b), the region between *δ* 3.0 and 5.5 ppm displayed the classic polysaccharide feature, and the signals in this area overlapped severely [70]. In the ^1^H NMR spectrum, HWE-AJBP1 identified 14 anomeric proton signals at chemical shifts of 5.37, 5.33, 5.29, 5.21, 5.17, 5.07, 5.03, 5.01, 4.90, 4.88, 4.87, 4.56, 4.55 and 4.45 ppm, and UAE-AJBP1 also identified 14 anomeric proton signals at chemical shifts of 5.37, 5.32, 5.30, 5.17, 5.07, 5.03, 5.00, 4.89, 4.89, 4.88, 4.87, 4.60, 4.55 and 4.44 ppm. There is broad consensus among researchers that the chemical shifts observed in the *δ*_H_ 5.0–4.4 ppm interval can be attributed to protons from both α-anomeric and β-anomeric protons [71], a finding that aligns with FT-IR analysis. At the same time, in ^13^C NMR spectrum, 14 abnormal carbon signals were also identified for HWE-AJBP1 and UAE-AJBP1. The anomeric carbon signals for HWE-AJBP1 were 109.83, 108.37, 107.51, 107.07, 103.65, 102.53, 100.36, 100.25, 99.03, 98.33, 98.04, 97.38, 95.88 and 92.13 ppm, while those for UAE-AJBP1 were 109.38, 108.75, 107.86, 107.43, 103.99, 103.00, 100.81, 100.73, 100.11, 98.39, 98.04, 97.27, 96.24 and 92.48 ppm, respectively. Moreover, Rha presents distinctive H-6 signals which can be readily differentiated from the signals of alternative carbohydrates in 1D spectra, generally appearing within the *δ*_H_ 1.2 ppm and *δ*_C_ 16 ppm intervals, as shown in Fig. 6c–d. Within this specified range, two abnormal signals were detected, and an analysis of monosaccharide composition indicated a high proportion of Rha. This implies that both types of glycosidic bonds (Rha) coexist in UAE-AJBP1 and HWE-AJBP1. And, a unique chemical shift noted at *δ*_C_ 170–176 ppm pointed to the presence of two kinds of uronic acids (GlcA and GalA) in both UAE-AJBP1 and HWE-AJBP1 [72]. This finding was corroborated by the analyses of chemical composition, monosaccharide composition, and FT-IR spectroscopy. At the same time, according to the measurement results of monosaccharide composition, the relative content of GalA was relatively high. The carbon signal observed in the vicinity of *δ*_C_ 175 ppm was attributed to GlcA, whereas the signal near *δ*_C_ 170 ppm was assigned to GalA. And, the typical uronic acid signal conventionally manifests around *δ*_C_ 175 ppm. Additionally, a pronounced signal density was identified within the *δ*_C_ 50–60 ppm range, and a distinct H proton signal was also detected at *δ*_H_ 3.72 ppm. This suggests that the presence of O-CH_3_ in the glycosidic bond types of GalA [73].

Specifically, through the integration of ^1^H NMR, ^13^C NMR, and the cross-peaks identified in the heterotopic region of HSQC spectra (Fig. 6e-h), the majority of signals detected by these NMR methods were predominantly found within the ^1^H range of 4.4–5.5 ppm and the ^13^C range of 90–110 ppm [74]. Given the similarity in their chemical shifts, this suggests that both UAE-AJBP1 and HWE-AJBP1 may contain the same 14 sugar residues. In HWE-AJBP1, the anomeric signals were identified with the following chemical shifts: *δ* 5.17/109.32, *δ* 5.38/108.94, *δ* 5.01/107.48, *δ* 5.08/107.04, *δ* 4.57/104.03, *δ* 4.44/102.72, *δ* 5.05/100.76, *δ* 4.89/100.34, *δ* 5.30/99.82, *δ* 4.87/98.85, *δ* 5.21/98.39, *δ* 4.90/97.76, *δ* 4.56/95.96 and *δ* 5.33/92.12 ppm. These signals were subsequently assigned to sugar residues labeled A through N, respectively. Additionally, the anomeric signals detected in UAE-AJBP1 exhibited chemical shifts at *δ* 5.17/109.26, *δ* 5.38/108.92, *δ* 5.01/107.61, *δ* 5.08/107.05, *δ* 4.59/103.87, *δ* 4.44/102.77, *δ* 5.06/100.94, *δ* 4.89/100.33, *δ* 5.31/99.71, *δ* 4.87/98.74, *δ* 5.05/97.93, *δ* 4.90/97.77, *δ* 4.56/95.90 and *δ* 5.33/92.10 ppm. These signals were subsequently designated as sugar residues A′, B′, C′, D′, E′, F′, G′, H′, I′, J′, K′, L′, M′, and N′, respectively. Drawing on the NMR data and pertinent literature findings, it can be deduced that residues A/A′, B/B′, C/C′, D/D′, E/E′, F/F′, G/G′, H/H′, I/I′, J/J′, K/K′, L/L′, M/M′, and N/N′ correspond to the following structures: →3,5)-*α*-L-Ara*f*-(1→ [75], *α*-L-Ara*f*-(1→ [29], →4)-*β*-D-Glc*p*-(1→ [32], →5)-*α*-L-Ara*f*-(1→ [76], →3)-*β*-D-Gal*p*-(1→ [77], →3,6)-*β*-D-Gal*p*-(1→ [29], *α*-D-Glc*p*-(1→ [78], →2,3)-*α*-D-GalA*p*-6-OMe-(1→ [29], →4)-*α*-D-GlcA*p*-(1→ [79], →2,4)-*β*-L-Rha*p*-(1→ [80], *α*-L-Rha*p*-(1→ [81], →4)-*β*-D-Xyl*p*-(1→ [82], *β*-D-Man*p*-(1→ [83] and *α*-D-Glc*p*-(4→ [84], respectively. Table 4 presents a comprehensive breakdown of the hydrocarbon signal assignments.

**Table 4:** ^1^H and ^13^C NMR chemical shifts of UAE-AJBP1 (A′-N′) and HWE-AJBP1 (A-N).

**Glycosyl residues**	**Chemical shifts of ^1^H/^13^C (ppm)**						
		**H1/C1**	**H2/C2**	**H3/C3**	**H4/C4**	**H5/C5**	**H6/C6**
→3,5)-*α*-L-Ara*f*-(1→	A	5.17/109.32	4.05/80.94	4.24/84.93	3.96/83.92	3.65/67.91	−
	A′	5.17/109.26	4.05/80.95	4.24/84.90	3.96/83.89	3.65/67.85	−
*α*-L-Ara*f*-(1→	B	5.38/108.94	3.80/81.33	4.05/76.25	4.00/83.93	3.56/62.50	−
	B′	5.38/108.92	3.97/81.38	4.05/76.47	4.01/83.93	3.56/62.48	−
→4)-*β*-D-Glc*p*-(1→	C	5.01/107.48	3.97/73.91	3.82/73.53	3.88/76.53	4.26/70.03	3.59/61.30
	C′	5.01/107.61	3.97/73.85	3.81/73.54	3.88/76.55	4.19/69.54	3.59/61.32
→5)-*α*-L-Ara*f*-(1→	D	5.08/107.04	4.23/81.51	4.14/76.36	4.13/82.32	3.81/66.81	−
	D′	5.08/107.05	4.23/81.50	4.13/76.41	4.13/82.34	3.81/66.79	−
→3)-*β*-D-Gal*p*-(1→	E	4.57/104.03	4.24/71.68	4.21/79.21	3.74/68.44	3.63/76.35	3.75/60.64
	E′	4.59/103.87	4.34/71.64	4.21/79.22	3.74/68.32	3.63/76.27	3.73/60.60
→3,6)-*β*-D-Gal*p*-(1→	F	4.44/102.72	4.18/71.95	4.33/78.81	3.84/68.77	3.20/75.92	4.05/70.12
	F′	4.44/102.77	4.24/71.77	4.32/78.68	4.06/68.47	3.32/76.07	4.26/70.01
*α*-D-Glc*p*-(1→	G	5.05/100.76	3.57/72.06	3.76/72.35	3.56/69.39	3.68/70.55	3.65/60.86
	G′	5.06/100.94	3.57/71.97	3.69/72.22	3.56/69.44	3.68/70.53	3.65/60.73
→2,3)-*α*-D-GalA*p*-6-OMe-(1→	H	4.89/100.34	4.09/77.60	4.18/79.86	3.34/69.50	3.43/75.74	−/170.75
	H′	4.89/100.33	4.09/77.56	4.18/79.99	3.33/69.50	3.40/75.66	−/170.57
→4)-*α*-D-GlcA*p*-(1→	I	5.30/99.82	3.88/73.34	3.71/73.56	3.58/76.89	3.82/69.49	−/173.50
	I′	5.31/99.71	3.87/73.24	3.71/73.54	3.58/76.95	3.81/69.41	−/176.62
→2,4)-*β*-L-Rha*p*-(1→	J	4.87/98.85	3.58/78.15	3.92/71.51	3.26/82.72	3.39/69.24	1.20/16.59
	J′	4.87/98.74	3.59/78.13	3.92/72.43	3.26/82.74	3.39/69.26	1.21/16.92
*α*-L-Rha*p*-(1→	K	5.21/98.39	3.76/71.14	3.88/71.70	3.98/72.40	3.73/69.79	1.17/16.11
	K′	5.05/97.93	3.75.71.21	3.88/71.72	3.98/72.51	3.73/69.79	1.16/16.45
→4)-*β*-D-Xyl*p*-(1→	L	4.90/97.76	3.93/70.41	3.99/70.68	4.01/76.88	3.18/62.48	nd
	L′	4.90/97.77	3.91/70.56	4.05/70.10	4.00/76.88	3.48/62.46	nd
*β*-D-Man*p*-(1→	M	4.56/95.96	3.44/71.78	4.12/73.72	3.47/73.59	3.72/66.79	3.51/59.69
	M′	4.56/95.90	3.39/71.95	4.13/73.82	3.47/73.52	3.72/66.88	3.51/59.69
*α*-D-Glc*p*-(4→	N	5.33/92.12	3.28/73.32	3.18/73.28	3.93/76.71	3.91/69.14	3.40/59.96
	N′	5.33/92.10	3.28/73.24	3.17/73.20	3.93/76.65	3.91/69.09	3.40/59.88

More importantly, there were certain differences in the 1D and 2D NMR spectra of UAE-AJBP1 and HWE-AJBP1, apart from the same parts analyzed above. It can be seen from the ^13^C spectra of HWE-AJBP1 (Fig. 6c) and UAE-AJBP1 (Fig. 6d) that the relative heights of the peaks were significantly different, which means that the relative proportions of various sugar residues were different. Drawing on the preceding analysis, this research indicated that while the mechanical and cavitation forces exerted during ultrasonic treatment did not alter the types of glycosidic bonds, they did lead to changes in the relative proportions of these bond types. The occurrence of this phenomenon may be mainly due to the unique structure of polysaccharides in *A*. *julibrissin* bark and the ordered midpoint chain scission caused by ultrasound.

#### 3.2.6 Conformation analysis of UAE-AJBP1 and HWE-AJBP1

**Fig. 7:**
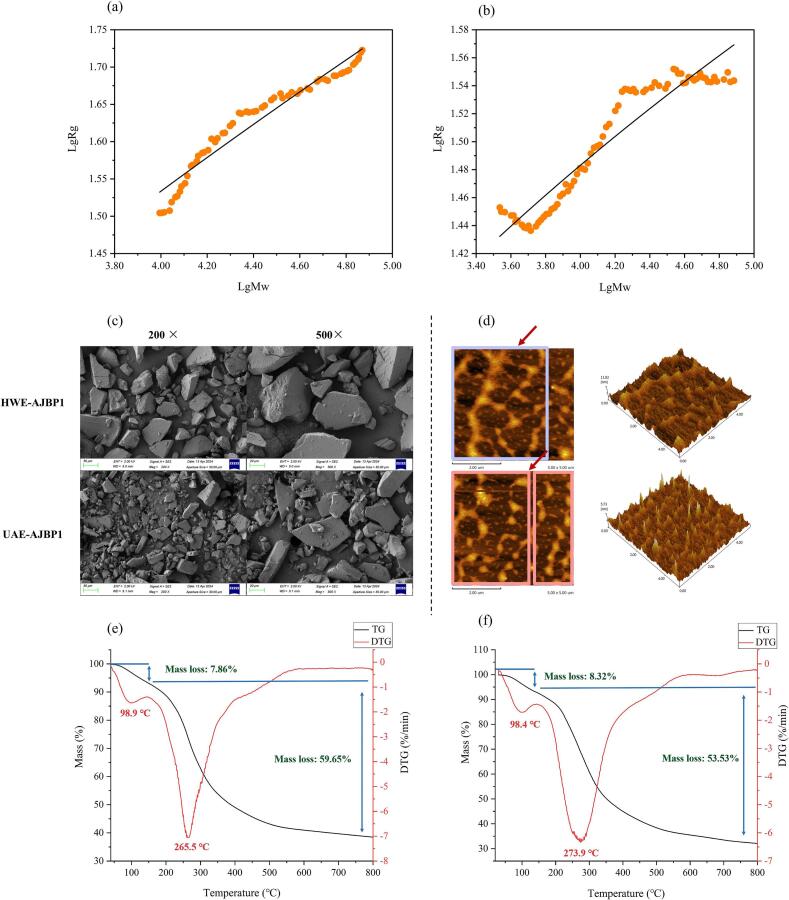
Chromatograms of chain conformation of HWE-AJBP1 (a) and UAE-AJBP1 (b) in NaNO_3_ aqueous solution; The microscopic morphology of HWE-AJBP1 and UAE-AJBP1: scanning electron micrographs (c) and atomic force microscopy images (d); TG and DTG curves of HWE-AJBP1 (e) and UAE-AJBP1 (f).

A multitude of research endeavors have indicated that the physicochemical characteristics and biological functionalities of polysaccharides are influenced not solely by their primary structures, but also by the conformations of their chains [54]. Upon dissolution in a solution, polysaccharides could adopt a range of conformations, including spherical or spherical-shell shapes, random coils, semi-rigid chains, oblate ellipsoidal, rod-like chains, and semi-flexible structures, among others. According to the study referenced in [85], ultrasonic degradation serves as an efficient and promising approach for investigating the structural–functional correlations of polysaccharides by altering their chain conformations. Meanwhile, the precise effects of ultrasound-induced advanced structural modifications on the structural properties, functions, and biological activities of polysaccharides remain inadequately understood. In Fig. 7a–b, the correlation between the Rg and the Mw of UAE-AJBP1 and HWE-AJBP1 within a NaNO_3_ solution was presented, and this correlation can be fitted by Rg = KMw**^β^**. In equation, K stands for an empirical constant, while β serves as a conformational parameter that indicates the spatial configuration of polymer molecules. Generally, β values falling within the ranges of 0 to 0.3, 0.5 to 0.6, and exactly 1.0 correspond to spherical, flexible chain, and rigid rod-like conformations of polysaccharide solutions, respectively [14,85]. The double logarithmic plot of Mw versus Rg was applied to calculate the K and β. By fitting the curve using nonlinear fitting, the exponent β values of UAE-AJBP1 and HWE-AJBP1 were obtained to be 0.28 and 0.59, respectively. HWE-AJBP1 exhibited a flexible chain conformation, while UAE-AJBP1 exhibited a spherical structure. According to reports, the spherical structure endows polysaccharides with good biological activity, such as anti-tumor activity, anti-aging effects, iron-chelating and free radical scavenging abilities [[86], [87], [88]]. This will enable UAE-AJBP1 to be better developed and applied in the fields of food and medicine.

#### 3.2.7 Microscopic morphology analysis of UAE-AJBP1 and HWE-AJBP1

Scanning electron microscopy (SEM) is highly acknowledged as a premier method for scrutinizing the structural morphology of macromolecules, encompassing aspects such as their size, shape, and porosity characteristics. Polysaccharides typically exhibit a more intricate solid shape geometry compared to nucleotides and proteins. The morphological characteristics of polysaccharides exert a significant influence on their solubility, release rate, and viscosity, consequently impacting their functional attributes. In the present study, SEM was employed to characterize the microstructural and surface morphological features of the polysaccharides, with a thorough investigation into the effect of ultrasonic degradation on HWE-AJBP1. SEM further elucidates the morphological properties of UAE-AJBP1 and HWE-AJBP1. As illustrated in Fig. 7c, notable disparities in size and shape were observed on the surfaces of HWE-AJBP1 and its degraded polysaccharides (UAE-AJBP1). At 100 × and 500 × magnification, the HWE-AJBP1 surface exhibited a uniform morphology, featuring a smooth, dense, and block-like morphology with well-defined boundaries. This phenomenon might be attributed to the hot water extraction process. Some polysaccharides have a propensity to develop longer polymer chains, and this subsequently leads to a smoother and more homogeneous surface morphology [24]. The underlying reason lies in the fact that plant polysaccharides contain a relatively large quantity of hydroxyl and carbonyl groups. These groups amplify the intermolecular and intramolecular interactions among polysaccharide molecules, thus leading to polysaccharide aggregation [89]. In contrast, after ultrasonic treatment, the SEM images of the UAE-AJBP1 fractions subjected to ultrasound revealed a transformation from the initial large block-like morphology into smaller block-like morphology. Excitingly, UAE-AJBP1 exhibits a more complex surface morphology, covered not only with smaller block-like morphology but also with many round particles (tiny spherical structure) of varying sizes. This outcome is also in strong alignment with the results of the conformation analysis, providing further validation for the accuracy of the conformations of UAE-AJBP1 and HWE-AJBP1. These findings may stem from the intense cavitation effects, turbulence shear, and instantaneous pressure decreases encountered during the processing. Moreover, the ultrasonic energy was adequate to disrupt covalent bonds, like the glycosidic bonds linking polysaccharide units, which is in line with the results obtained from Mw measurement and analysis [90].

Atomic Force Microscopy (AFM) serves as a method for examining polymers at the sub-nanometer scale and holds equally valuable in characterizing the morphology of nanostructures, as well as the spherical or linear arrangements of polysaccharides. It offers the benefit of enabling the detection and visualization of polysaccharides morphologies through the intermolecular force acting between the probe and the sample [91]. This study further employed the AFM technique to analyze the molecular chain conformations of UAE-AJBP1 and HWE-AJBP1, offering a certain scientific foundation for exploring the structure–activity relationship and ultrasonic treatment mechanism of polysaccharides. In Fig. 7d, HWE-AJBP1 molecules displayed aggregate and network structure, suggesting that HWE-AJBP1 exhibited distinctively long primary chains along with branched structures. The clustering of polysaccharide molecules can likely be ascribed to strong intramolecular and intermolecular forces stemming from hydrogen bonds and electrostatic interactions [92]. Following ultrasonic treatment, the aggregates within UAE-AJBP1 exhibited a reduction in size, however, the sugar chains preserved numerous branches and a cross-linked network structure. Given that fuzzy ends and branching sites were observable, the interaction among numerous polysaccharide chains was substantiated, which aligns with previous findings in other linear polysaccharides such as *Pericarpium Citri Reticulatae* polysaccharides [57] and *Houttuynia cordata* polysaccharides [44]. However, there was a decrease in chain length alongside an increase in the emergence of spherical structures. And UAE-AJBP1 does not exist in the form of a single polysaccharide molecule, mainly due to its presence of HG structure (homogalacturonan regions) during the degradation process, exhibiting relatively high flexibility [57]. This flexibility leads to molecular bending, folding, and curling of the flexible chain, resulting in its spherical structure. Moreover, from a three-dimensional perspective, it was evident that numerous bars formed and aggregated into relatively loose and tight structures in HWE-AJBP1 and UAE-AJBP1, respectively. These structures bore a resemblance to the appearance of stone forests and were distributed in a random fashion across the surfaces of HWE-AJBP1 and UAE-AJBP1. It is worth mentioning that the nanoscale height significantly decreases after ultrasonic treatment, which typically represented shorter branch lengths, as we can see in AFM plan view. This corroborated the findings from the Mw determination, SEM examination, and conformational analysis.

SEM images prominently illustrate significant changes in particle dimensions, form, and clustering, whereas AFM depictions offer nanoscale morphological features and chain conformation. Analysis using these two microscopy methods demonstrated that UAE-AJBP1 possessed more complex, smaller block-like morphology with tiny spherical structures compared to HWE-AJBP1, which had smoother, dense, and block-like morphology. The primary factor contributing to the morphological disparities between HWE-AJBP1 and UAE-AJBP1 lay in ultrasonic processing. This ultrasonic processing has the capability to induce degradation, resulting in the breaking of glycosidic bonds and the generation of smaller molecular segments [93]. Based on the above analysis, the midpoint degradation caused by ultrasound leaded to the formation of small spherical conformations in this small molecule fragment, which no longer extended into a chain like conformation due to its possible presence of HG structure and being a flexible chain. However, the conformation of UAE-AJBP1 was a combination of flexible chain (precious few) and spherical conformation, which may have been due to the limited ultrasound power (201 W) used for extraction in this study. A minor deviation was observed in the conformational analysis, which was understandable. The reason behind this was the scarcity of chain-like conformations due to ultrasonic degradation, and they either went undetected or were masked by the sensitivity of the detection method.

#### 3.2.8 TGA analysis of UAE-AJBP1 and HWE-AJBP1

It is widely acknowledged that structural elements such as Mw, crystallinity, and fiber alignment exert a considerable influence on the thermal degradation process [94]. Attempting to analyze the effect of structural changes in UAE-AJBP1 treated with ultrasound on thermal stability through thermogravimetric analysis. In addition, thermal stability serves as a crucial property for materials with potential biological applications, particularly given the possible of sterilization via heat treatment. Understanding the thermal characteristics of *A. julibrissin* bark polysaccharides is also vital for its appropriate industrial utilization in the future.

As illustrated in Fig. 7e–f, both UAE-AJBP1 and HWE-AJBP1 demonstrated two distinct weight loss phases within the temperature range of 30°C to 800°C. Moreover, UAE-AJBP1 and HWE-AJBP1 exhibited similar trends. In the initial phase, spanning the temperature range from approximately 30°C to 98°C, the mass reduction observed in both UAE-AJBP1 and HWE-AJBP1 samples was linked to the physically adsorbed water and structural water. Polysaccharide encompass a substantial quantity of hydrophilic groups, which enable them to take up a certain amount of water as the temperature increases [95]. Additionally, the polysaccharides by us prepared remarkably strong water absorbing capabilities, even under room temperature circumstances. Given that crystalline regions within polysaccharide structures typically lack the capacity for water absorption, the water that has been taken up is associated with the amorphous regions present in the polysaccharide architecture. It is worth noting that UAE-AJBP1 showed the highest weight loss at this stage (8.32 %), indicating its outstanding water-holding capacity, which may be more suitable for development and application in fields such as food and materials.

The second phase involved the breakdown of UAE-AJBP1 and HWE-AJBP1 chains, along with the disruption of both intermolecular and intramolecular hydrogen bonds [17]. This occurrence is likely attributable to the degradation initiated by the thermal decomposition of the polysaccharide, and it was characterized by the onset of weight loss and the oxidation temperature. Significantly, this process resulted in a marked and swift weight reduction of approximately 55 %, suggesting considerable harm to the structural integrity of UAE-AJBP1 and HWE-AJBP1. Notably, this stage the weight loss of the UAE-AJBP1 (53.53 %) was less than that of HWE-AJBP1 (59.65 %), and final residual mass of 32.49 % (HWE-AJBP1) and 38.15 % (UAE-AJBP1). The findings provided evidence supporting the idea that ultrasonic treatment notably improves the thermal stability of *A. julibrissin* bark polysaccharides, an effect likely stemming from the structural optimization of these polysaccharides induced by ultrasound.

#### 3.2.9 Triple helix structure and CD spectroscopy analysis of UAE-AJBP1 and HWE-AJBP1

**Fig. 8:**
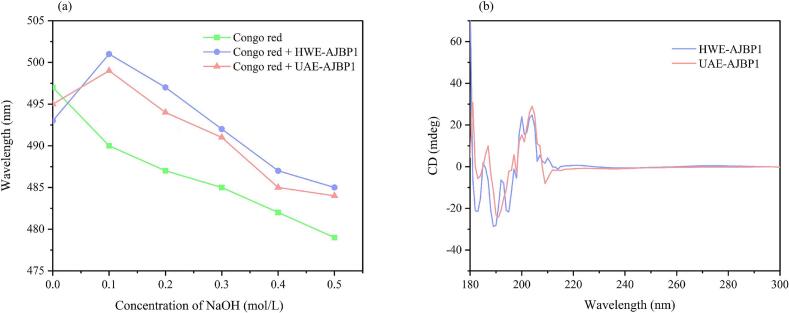
Maximum absorption wavelength of Congo red complex at different concentrations of NaOH (a), and CD spectra (b).

In comparison to nucleins and proteins, polysaccharides have always confronted major challenges related to structural complexity, including the determination of the triple helix conformation. In addition, numerous reports have highlighted that many triple helix polysaccharides offer diverse health advantages, such as anticancer, immunomodulatory, and antioxidant activities [96]. Research findings further suggest that the triple helix conformation serves as a critical factor contributing to the reported health-related effects [96]. In investigations of the polysaccharide triple helix conformation, Congo red plays a significant role. Upon exposure to a relatively high-concentration alkaline solution, the triple helix structure undergoes denaturation. When a high density of charges is introduced along every strand comprising the triple helix, it gives rise to a scenario where electrostatic repulsion takes place among these strands [97]. The resulting repulsive forces disrupt the triple-helix assembly, and higher alkaline concentrations accelerate conversion to alternative conformations (double helix, single helix, or random coil) [97]. In other words, when the maximum absorption wavelength of the Congo red-polysaccharide complex is compared for polymers possessing a triple-helix structure, a successive red-shift followed by a blue-shift phenomenon is observed [98]. In Fig. 8a, the spectral profiles of Congo red and its complexes with UAE-AJBP1 and HWE-AJBP1 were presented, recording their maximum absorption wavelength. It was worth noting that the maximum absorption wavelength exhibited both red and blue shift phenomenon successively. Therefore, it was speculated that UAE-AJBP1 and HWE-AJBP1 had a triple helix structure.

Circular dichroism (CD) spectroscopy is commonly utilized for investigating the triple helix structure and the conformational alterations of biopolymers in solution. When the light traverses the photoactive medium and the wavelengths of the two polarized light beams lie within the absorption range of the photoactive chromophore, the differing light absorption characteristics result in unequal reductions in the amplitudes of the two transmitted beams [99]. As a consequence, the superposition of these transmitted light beams gives rise to an elliptically polarized state. Recording ellipticity θ for different wavelengths leads to the acquisition of the circular dichroism spectrum. This study compared the conformational changes of UAE-AJBP1 and HWE-AJBP1 before and after ultrasound by recording the differences in optically active chromophores, and confirmed the existence of their triple helix conformation. During the analytical procedure, while the polysaccharide structure does not precisely conform to the α spiral or β folding conformations observed in proteins, it can generally be categorized as a disordered chain, an extended rigid chain, or a helical chain [100]. Current research has demonstrated that the characteristic CD spectral bands of polysaccharides are usually located within the range of 190 to 240 nm [100]. As shown in Fig. 8b, UAE-AJBP1 and HWE-AJBP1 exhibited positive and negative Cotton effects between 190–240 nm [101], indicating that UAE-AJBP1 and HWE-AJBP1 existed in aqueous solution in helical form, a finding that aligns with the analysis outcomes from the Congo red experiment. The development of these active triple-helix polysaccharides plays a role in advancing research and fostering innovation in sustainable “green” biopolymers within the fields of food science and life sciences [102]. In addition, CD spectroscopy once again confirmed that ultrasound treatment changed the conformation of polysaccharides in *A. julibrissin* bark. Conformational changes affect chromophore orientation, static field contribution, and polarization sensitivity, altering spectral intensity [103]. As observed in Fig. 8b, ultrasonic intervention enhanced spectral intensity, potentially attributable to polysaccharide degradation that increased the number of terminal carboxyl groups (–COOH). Consequently, this augmented both intra- and intermolecular hydrogen bonding [57,103], potentially accounting for the self-coiling and folding behavior observed in UAE-AJBP1 molecules. FT-IR spectrometer, monosaccharide composition and chemical composition analysis can further support this phenomenon.

### 3.3 Biological activity

**Fig. 9:**
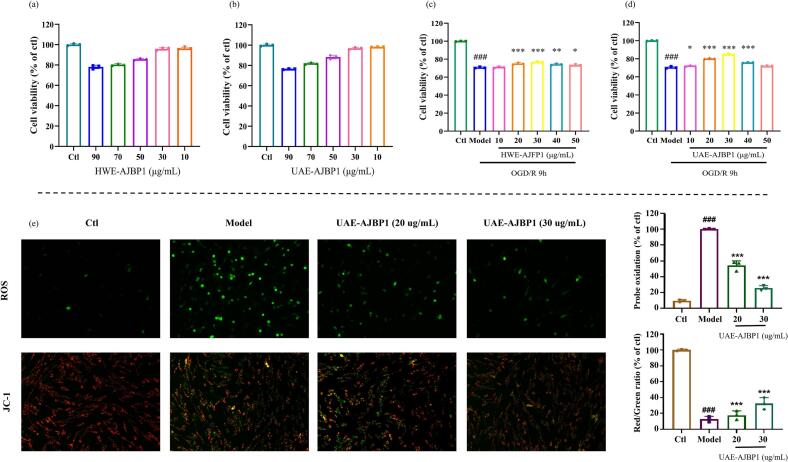
Cytotoxicity detection of HWE-AJBP1 (a) and UAE-AJBP1 (b) on PC12 cells; The protective effects of HWE-AJBP1 (c) and UAE-AJBP1 (d) on PC12 cells induced by OGD/R model; (n = 3; # compared with the Ctl group, ### p < 0.001; * compared with the Model group, *** p < 0.001).

The cytotoxic impact of UAE-AJBP1 and HWE-AJBP1 on PC12 cells was evaluated using the CCK-8 assay. The findings demonstrated that PC12 cell viability exceeded 85 % upon exposure to concentrations of these two polysaccharides spanning from 10 to 50 µg/mL (as depicted in Fig. 9a–b). This result suggested that UAE-AJBP1 and HWE-AJBP1 do not exhibit cytotoxic properties within this concentration range. Following this, the cytoprotective actions of UAE-AJBP1 and HWE-AJBP1 on PC12 cells were assessed across the 10–50 µg/mL concentration spectrum, utilizing the Oxygen Glucose Deprivation/Reperfusion (OGD/R) model as a basis (Fig. 9c–d). UAE-AJBP1 demonstrated significantly superior cellular protection versus HWE-AJBP1 (P < 0.001), a difference potentially resulting from ultrasonication-induced polysaccharide structure changes. As one of the most suitable technologies for industrial production, UAE also plays a unique and crucial role in the research of polysaccharides from *A. julibrissin* bark using ultrasound. According to previous reports, apoptosis, mitochondrial dysfunction and oxidative stress mechanisms, which contribute to the pathophysiology of ischemic stroke, and ultimately result in tissue infarction and neuronal death [104,105]. For this purpose, JC-1 staining and DCFH-DA staining were used to determine the effect of UAE-AJBP1 on ODG/R-induced oxidative stress. The experimental results indicate that UAE-AJBP1 significantly attenuated oxidative stress and reinstated mitochondrial membrane potential (Fig. 9e). These results indicate that the neuroprotective effects of UAE-AJBP1 may be mediated through multiple pathways, including anti-oxidative stress, mitochondrial protection, and anti-apoptosis.

The bioactivity of different polysaccharides is closely associated with sugar structural configurations and conformations [106]. That is to say, the development potential of UAE-AJBP1 in the field of stroke is attributed to the changes of polysaccharide structure through ultrasound treatment. The structural study of the UAE-AJBP1 and HWE-AJBP1 mentioned above indicates that they share similar functional groups, monosaccharide compositions, and both have triple helix conformation, but differ in chemical compositions, monosaccharide composition molar ratios, MW, glycosidic bond content, microstructures, thermal stability and chain conformation. All of these factors suggested that they may exert different PC12 cell protective effects based on OGD/R model. Charged groups, such as acidic groups, dictate polysaccharide neutrality or acidity in molecular structures [107]. Here, both UAE-AJBP1 and HWE-AJBP1 contain uronic acid, suggesting potential bioactivity, however, variations in uronic acid content and possible connection modes likely underlie differences in their activity. In addition, the structural characteristics of polysaccharides, particularly the proportion of monosaccharides within the sugar ring, play a crucial role in determining their biological properties [108]. Meanwhile, the characteristics of glycosidic bonds within polysaccharides, encompassing their types, content, configurations, and positions, exert a notable impact on their biological activities [109]. In the current study, both UAE-AJBP1 and HWE-AJBP1 possess identical 8 monosaccharide compositions and 14 glycosidic bond types, with different molar ratios. It can be inferred that relatively speaking, high levels of Man, Rha, GalA, and Gal, as well as low levels of Glc and → 4)-*β*-D-Glc*p*-(1→, may exert better neuroprotective effects. Moreover, the MW of polysaccharides is one of the important influencing indicators of their biological activity [110]. According to this experiment, preparing *A. julibrissin* bark polysaccharides with an MW range of 1.475 × 10^5^-2.617 × 10^5^ kDa indicated neuroprotective effects, however, lower MW fractions appear to exhibit stronger activity. Finally, the physicochemical properties and biological functions of polysaccharides are not only influenced by their primary structure, but also by their chain conformation and microstructures. The results of this study suggested that smaller block-like morphology and spherical conformations might endow polysaccharides with relatively good neuroprotective effects based on the OGD/R model.

### 3.4 The possible mechanism of ultrasonic degradation of *a. Julibrissin* bark polysaccharides

**Fig. 10:**
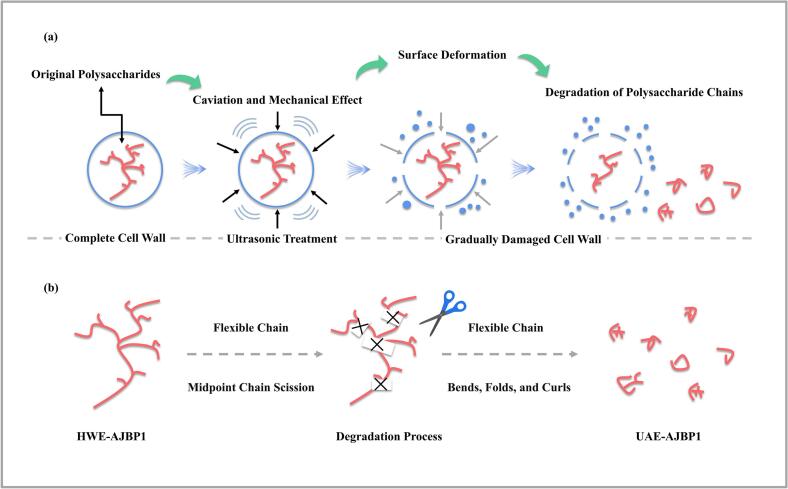
Possible degradation mechanism of polysaccharides from *A. julibrissin* bark under ultrasonic treatment.

Integrating the findings from degradation kinetics and structural analysis, the proposed mechanism for the ultrasonic degradation of UAE-AJBP1 is illustrated in Fig. 10. Generally, the two primary mechanisms involved in the modification of plant polysaccharides through ultrasonic treatment are mechanical bond breaking and cavitation. For the extraction rate mentioned in this study, during mechanical bond breaking and cavitation processes, due to the high motion acceleration and thermal effects caused by ultrasound, they generate strong and rapid mechanical motion on the cell wall of HWE-AJBP1. They will disrupt cell integrity, modify tissue architecture, promote the entry of water molecules, thereby increasing the extraction rate (Fig. 10a). On the other hand, the mechanical bond severing and cavitation phenomenon induced by ultrasonic waves can lead to the rupture of chemical bonds in the main chain of polysaccharides, subsequently triggering the self-degradation of polysaccharides from UAE-AJBP1. Meanwhile, the bubbles formed due to ultrasonic cavitation underwent rapid collapse, generating significant shear forces. These forces facilitated the breakdown of polysaccharide chains having a higher Mw, causing them to preferentially degrade into stable small molecular fragments (Midpoint scission). This small molecule fragment, due to its flexible chain and HG structure, bends, folds, and curls, resulting in the spherical conformation of UAE-AJBP1 (Fig. 10b). In particular, due to the UAE-AJBP1 having triple helix structure, which increases molecular rigidity, it is speculated that the spherical conformation of UAE-AJBP1 is not as tight.

More specifically, ultrasound treatment as an important physical intervention method, this study explored the effects of ultrasound treatment on the physicochemical properties and chain conformation of *A. julibrissin* bark polysaccharides from multiple dimensions using ultrasound treatment conditions of 50 min and 201 W. The experimental results showed that ultrasound treatment had a significant effect on the chemical composition of *A. julibrissin* polysaccharides under this treatment condition (50 min and 201 W), and changed the relative content of its characteristic functional groups. Meanwhile, ultrasound treatment leads to dynamic adjustment of the molar ratio of monosaccharide composition, which is directly related to the biological activity and functional properties of polysaccharides. Furthermore, ultrasonic degradation significantly reduced the Mw distribution range of HWE-AJBP1 and induced its chain conformation to transition from a spherical conformation, which has profound effects on the solubility, viscosity, and interaction with biomolecules of polysaccharides. Additionally, ultrasound treatment also altered the relative content of glycosidic bond types, which is an important basis for the structural diversity and functional complexity of polysaccharides. Although the ultrasound power (201 W) in this study was relatively small, this may be due to the special structure of polysaccharides from *A. julibrissin* bark. At the microscopic morphological level, ultrasound treatment causes significant changes in the microscopic morphology of HAE-AJBP1, which are closely related to its macroscopic physical and chemical properties. Moreover, ultrasound treatment significantly affects the thermal stability, manifested as the increase in final remaining mass, which is crucial for understanding the stability of polysaccharides in processing, storage, and application processes. It can be seen that ultrasound treatment has a certain degree of influence on both the primary and advanced structures of HAE-AJBP1 (the possible reasons for these phenomena are detailed in the analysis and discussion of the above results). Through multidimensional analysis and comparison, these structural changes collectively affect the exposure of active sites.

It is worth mentioning that in this study, a spherical conformation of the polysaccharides fraction UAE-AJBP1 was prepared under ultrasound conditions for 50 min at 201 W. The reason worth mentioning is that the recent review [54] and research articles [[111], [112], [113]] of ultrasound degradation of polysaccharides have shown that the majority of research on the mechanism of ultrasound degradation has focused on conventional aspects such as molecular weight, monosaccharide composition, and chemical bonds. However, there are relatively few in-depth studies on the chain conformation and advanced structure of polysaccharides treated with ultrasound. In depth analysis of this phenomenon reveals that its root cause mainly lies in the limitations of the current technology used to explore the advanced structure of polysaccharides, which makes it difficult to accurately capture the subtle changes in polysaccharide structure during ultrasonic treatment. In the future, we will strive to develop precise technologies for exploring the advanced structures of polysaccharides, fully revealing the comprehensive impact mechanism of ultrasound treatment on polysaccharide structures, and promoting the widespread application of ultrasound technology in the field of polysaccharides.

## 4 Conclusions

In order to explore more comprehensively ultrasound's impact on *Albizia julibrissin* Durazz. bark polysaccharide extraction yield and structure, this study compared HWE and UAE for obtaining polysaccharides (HWE-AJBP1 and UAE-AJBP1). UAE increased extraction yield by 2 % versus HWE, with optimal parameters at 50 min, 70 ℃, 30 mL/g, and 201 W. For industrial production, efficient extraction is crucial. In addition, this study found that ultrasound can alter the chemical composition, characteristic group content, monosaccharide composition molar ratio, molecular weight, and even relative content of glycosidic bond types of polysaccharides from *A. julibrissin* bark. Interestingly, the ultrasound treatment followed the midpoint chain scission model and transformed HWE-AJPP1 from a flexible chain conformation to UAE-AJPP1 exhibiting a spherical structure. Ultrasound also altered HWE-AJPP1′s tight big block-like morphology, improving its thermal stability. Meanwhile, it was exciting to note that the protective effect of UAE-AJBP1 on PC12 cells based on the OGD/R model was higher than that of HWE-AJBP1. The finding that ultrasound can induce structural changes in polysaccharides gives new understandings about the structure–activity relationship and structural modification of polysaccharides. This discovery encourages the application of ultrasound technology in the polysaccharide area and may assist in exploring and creating new polysaccharide-based foods, materials, and products.

## CRediT authorship contribution statement

**Yuanqi Duan:** Writing – original draft, Methodology, Formal analysis, Data curation. **Yanan Liu:** Methodology, Investigation. **Man Li:** Visualization, Investigation. **Yajie Liu:** Software, Methodology. **Jiayu Gu:** Visualization, Software. **Wei Zhou:** Validation, Supervision, Project administration. **Jinfeng Sun:** Supervision, Project administration, Conceptualization. **Zhengyu Hu:** Writing – review & editing, Project administration, Methodology. **Mei Jin:** Project administration, Conceptualization. **Gao Li:** Project administration, Conceptualization.

## Declaration of competing interest

The authors declare that they have no known competing financial interests or personal relationships that could have appeared to influence the work reported in this paper.
